# Stiffening Matrix Induces Age‐Mediated Microvascular Phenotype Through Increased Cell Contractility and Destabilization of Adherens Junctions

**DOI:** 10.1002/advs.202201483

**Published:** 2022-06-03

**Authors:** Rahel Schnellmann, Dimitris Ntekoumes, Mohammad Ikbal Choudhury, Sean Sun, Zhao Wei, Sharon Gerecht

**Affiliations:** ^1^ Department of Chemical and Biomolecular Engineering Johns Hopkins University Baltimore MD 21218 USA; ^2^ The Institute for NanoBioTechnology Physical Sciences‐Oncology Center Johns Hopkins University Baltimore MD 21218 USA; ^3^ Department of Mechanical Engineering Johns Hopkins University Baltimore MD 21218 USA; ^4^ Department of Materials Science and Engineering Johns Hopkins University Baltimore MD 21218 USA; ^5^ Department of Biomedical Engineering Johns Hopkins University Baltimore MD 21218 USA; ^6^ Department of Biomedical Engineering Duke University Durham NC 27708 USA

**Keywords:** collagen, disease model, extracellular matrix mechanics, hydrogel, induced pluripotent stem cells, vascular aging

## Abstract

Aging is a major risk factor in microvascular dysfunction and disease development, but the underlying mechanism remains largely unknown. As a result, age‐mediated changes in the mechanical properties of tissue collagen have gained interest as drivers of endothelial cell (EC) dysfunction. 3D culture models that mimic age‐mediated changes in the microvasculature can facilitate mechanistic understanding. A fibrillar hydrogel capable of changing its stiffness after forming microvascular networks is established. This hydrogel model is used to form vascular networks from induced pluripotent stem cells under soft conditions that mimic young tissue mechanics. Then matrix stiffness is gradually increased, thus exposing the vascular networks to the aging‐mimicry process in vitro. It is found that upon dynamic matrix stiffening, EC contractility is increased, resulting in the activation of focal adhesion kinase and subsequent dissociation of *β*‐catenin from VE‐Cadherin mediated adherens junctions, leading to the abruption of the vascular networks. Inhibiting cell contractility impedes the dissociation of *β*‐catenin, thereby preventing the deconstruction of adherens junctions, thus partially rescuing the age‐mediated vascular phenotype. The findings provide the first direct evidence of matrix's dynamic mechano‐changes in compromising microvasculature with aging and highlight the importance of hydrogel systems to study tissue‐level changes with aging in basic and translational studies.

## Graphical abstract

To mimic the aging matrix, a fibrillar hydrogel system capable of changing its stiffness after the formation of microvascular networks is presented. Upon dynamic matrix stiffening, endothelial cell contractility increases, resulting in dissociation of *β*‐catenin from vascular endothelial‐cadherin mediated adherens junctions, thus destructing the vascular networks. This is the first direct evidence of aging matrix regulation of tissue microvascular network phenotype and possible therapeutic targets.

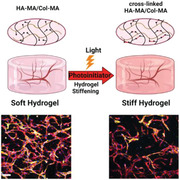

## 1 Introduction

Age is a significant risk factor for cardiovascular diseases such as atherosclerosis, hypertension, and ischemia.^[^ ^1^, ^2^ ^]^ However, aging not only affects large arteries and veins but was also shown to impair the function of tissue microvasculature. Several studies have shown that the density of tissue microvasculature declines dramatically with increasing age,^[^ ^3^, ^4^, ^5^, ^6^, ^7^ ^]^ contributing to the failure of vascular recovery in aged organs.^[^ ^8^ ^]^ Especially, vascular density and functionality in the kidney decrease strongly with aging.^[^ ^9^, ^10^, ^11^, ^12^ ^]^ The kidney is not the only organ showing reduction of vascular density and dysfunction upon aging. Microvasculature density is also reduced in the aged brain,^[^ ^13^, ^14^, ^15^ ^]^ retina,^[^ ^16^ ^]^ skin,^[^ ^17^ ^]^ and skeletal muscle.^[^ ^18^, ^19^ ^]^ In addition to reduced density, microvessels in aged tissues show altered morphology and impaired vascular organization than capillaries in young tissues.^[^ ^8^, ^17^ ^]^ Various events such as chronic inflammation, diabetes, and high blood pressure, have been identified to contribute to the observed loss and dysfunction of the tissue microvasculature during aging.^[^ ^20^, ^21^, ^22^ ^]^ Additionally, a common theory is that the angiogenic process is strongly impaired in aged tissues, thus partially explaining the lack of regenerative capacity and the decrease in capillaries to a certain extent.^[^ ^23^, ^24^ ^]^

Changes in the extracellular matrix (ECM) upon aging are well documented and could be associated with cellular dysfunction in large blood vessels.^[^ ^21^ ^]^ Specifically, in vivo evidence indicates that increased ECM stiffness in large vessels, such as arteries, might contribute to vascular dysfunction and diseases like hypertension and atherosclerosis.^[^ ^25^, ^26^, ^27^ ^]^ In these large vessels, an increase in collagen deposition in the vessels’ walls and the subsequent changes in their mechanical properties can have fatal effects on endothelial cell (EC) function via mechano‐sensitive signaling pathways.^[^ ^28^, ^29^ ^]^ Previous studies further suggested that an increase in ECM stiffness influences the vascular morphology in tumor vessels leading to a leaky vascular phenotype.^[^ ^30^ ^]^ Indeed, ECs are mechanosensitive, responding to both shear stress and ECM mechanics.^[^ ^31^, ^32^, ^33^, ^34^, ^35^ ^]^ The ECM also plays an essential role in regulating new vascular network formation, including vasculogenesis and angiogenesis, through adhesion and mechanical signaling pathways in ECs.^[^ ^28^, ^29^, ^31^, ^32^, ^34^, ^36^ ^]^ Nonetheless, while there is some evidence that tissue stiffness gradually increases with aging,^[^ ^37^, ^38^ ^]^ there is limited understanding of if and how such changes in tissue stiffness affect microvasculature dysfunction observed during aging.

One of the main reasons for the lack of in‐depth knowledge of underlying pathways in microvascular aging is the limitation of physiological 3D systems to investigate tissue‐level aging in vitro. Most studies on the formation of new vascular networks focus on understanding the mechanism by which singular ECs or small EC‐aggregates respond to matrix properties that either allow or inhibit EC morphogenesis and network formation.^[^ ^36^, ^39^, ^40^ ^]^ Conversely, the study of responses to the aging ECM requires the exposure of an established 3D vascular network with continuous lumen to a stiffening matrix. Thus, in vitro 3D systems that support vascular morphogenesis and can mimic the dynamic changes in the tissue's mechanical properties are of increased importance to understand the contribution of ECM to tissue microvasculature aging and dysfunction and to elucidate the underlying signaling processes in ECs during aging fully.

We report that a decrease in the vasculature density in the aging kidney correlates with increased collagen deposition and tissue stiffness. We then utilize a fibril collagen‐based 3D hydrogel with the capability to gradually increase matrix stiffness “on‐demand” as a platform for the in vitro modeling of the aging ECM. Using this platform, we can form microvascular networks from stem cell‐derived ECs (iECs) in a hydrogel with relatively low elastic moduli (soft conditions), followed by a controllable increase in matrix stiffness surrounding the vascular networks. This platform allows us to analyze how a stiffening matrix impacts 3D microvascular phenotype and mechano‐sensitive pathway activation. Using this platform, we find that in response to the dynamic increase in matrix stiffness, microvascular phenotype changes with reduced vessel length, volume, and overall density, like the observed aging phenotypes in vivo. We further find that an increase in matrix stiffness leads to increased cell contractility, destabilizing adherens junctions via the dissociation of *β*‐catenin from vascular endothelial cadherin (VE‐Cadherin). Subsequently, we find that inhibition of cell contractility and focal adhesion kinase (FAK) activation allows rescue of the observed stiffness‐induced vascular phenotype and prevents the dissociation of *β*‐catenin from the adherens junctions. Overall, the presented work uses dynamic hydrogels to provide the first evidence of aging ECM regulation of tissue microvascular network phenotype and possible therapeutic targets.

## 2 Results

### 2.1 A Decrease in Vascular Density in Aged Kidneys Correlates with Increased Collagen Deposition and Tissue Stiffness In Vivo

**Figure 1:**
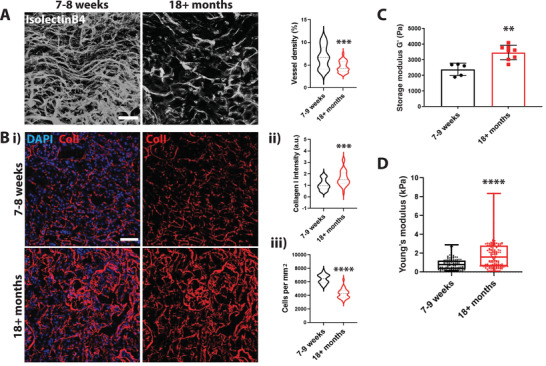
An increase in tissue stiffness correlates with a decrease in vascular density in aged kidneys. A) Representative 3D projection of cleared kidney tissue. Vasculature was visualized using isolectinB4. Scale bar is 50 µm. Quantification of vascular density. Vessel volume was quantified and normalized to the total volume of the imaged area. *N* = 4 animals with a total of 28 images. Bi) Representative images of Collagen I staining of a tissue section of aged and young mouse kidneys (DAPI in blue and Collagen I in red). Scale bar is 50 µm. ii) Quantification of relative collagen levels normalized to DAPI shows an increase in Collagen I deposition in aged kidneys iii) and a decrease in cell density. *N* = 3 kidneys with a total of 30 images were analyzed. C) Tissue rheology of young and aged mouse kidneys shows an increase in overall tissue stiffness with increasing age. *N* = 5 young mouse kidneys (7–9 weeks); *N* = 8 aged mouse kidneys (18+ months) were analyzed. D) AFM quantification of kidney cryosections of aged and young mice shows an increase in overall tissue stiffness with increasing age. Slices of 25 µm in thickness were analyzed. *N* = 3 mice and an average of 40 individual data points per section were measured. Significance levels were set at *p* ≤ 0.05, **p ≤ 0.01, ***p ≤ 0.001, and ****p ≤ 0.0001.

Aging was shown to significantly impact the morphology and density of the vasculature in various tissues.^[^ ^12^ ^]^ To correlate aging with vascular density and tissue ECM, we analyzed changes in kidney vasculature of aged mice (18+ months old) and young mice (7–9 weeks old). To determine the vessel density in aged tissue, we cleared kidney slices and stained blood vessels with the vascular specific marker isolectinB4. Aged kidneys showed a significantly reduced microvessel density (**Figure** **1**A) and a significant increase in kidney weight (Figure S1A, Supporting Information) compared to kidneys from young mice. Further analysis of the tissue structure revealed decreased cell density with increased Collagen I deposition in aged kidney mice compared to young kidneys (Figure 1Bi,Bii). Finally, we determined tissue stiffness using shear rheology and atomic force microscopy (AFM). We found an increase in overall stiffness ranging from an average of 2390 ± 398 Pa in young mouse kidneys to an average of 3474 ± 463 Pa in aged kidneys (Figure 1C,D; and Figure S1B, Supporting Information). Overall, we show that a change in mechanics of the aging tissue correlates with a decrease in microvascular density.

### 2.2 Methacrylate Hyaluronic Acid /Methacrylate Collagen I Hydrogels Recapitulate Dynamic Stiffening

To determine if dynamic tissue stiffness directly modulates capillary function and density, we sought to establish a hydrogel system that can support endothelial lumen formation and, subsequently, vascular network followed by a controllable, gradual increase in matrix stiffness mimicking the natural process of aging. We should note that vasculogenesis followed by vascular network formation occurs under soft hydrogel conditions.^[^ ^36^, ^41^, ^42^ ^]^ Thus, we used a stiffness range that supports network formation in hydrogels, focusing on recapitulating the twofold increase in stiffness observed within the aging kidney.

We mixed methacrylate hyaluronic acid (HA‐MA; Figure S2A, Supporting Information) with methacrylate collagen I (CoI‐MA) and allowed it to polymerize at 37 °C. We used Collagen I due to its fibrillary structure and importance in microvessel aging. We utilized methacrylate to allow dynamic stiffening. HA, shown to support vascular network formation in 3D hydrogels,^[^ ^40^, ^43^ ^]^ was added to increase the number of methacrylate sites allowing the optimization of a range of stiffness increase.

**Figure 2:**
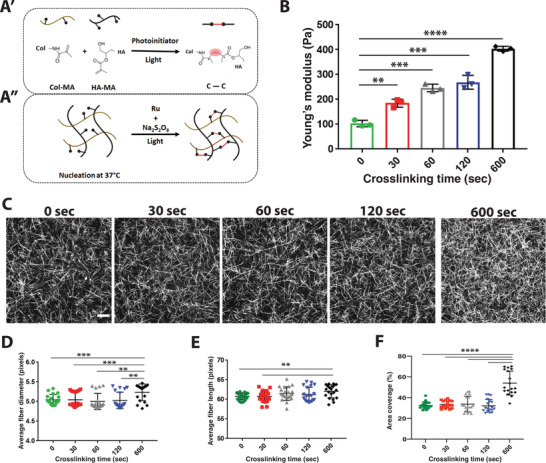
Characterization of dynamically stiffening collagen/HA hydrogel. A) Schematic of the HA‐MA /Col‐MA hydrogel system. B. AFM measurement of Young's modulus (Pa) of stiffening hydrogels. (*N* = 3 hydrogels per condition with ≈40 indentations per gel) C) Representative reflective confocal images of fiber structure of hydrogels after different time points of crosslinking. Scale bar is 20 µm. Analysis of D) average fiber diameter, E) average fiber length, and F) percent of fibers per area after different crosslinking times (*N* = 3 with 21 images analyzed per condition). Significance levels were set at *p* > 0.05, *p ≤ 0.05, **p ≤ 0.01, ***p ≤ 0.001, and ****p ≤ 0.0001.

After polymerization, the hydrogel was incubated with a Ruthenium photoinitiator and crosslinked using visible light (**Figure** **2**A). The viability assay demonstrated no impact of Ruthenium on iECs (Figure S2B, Supporting Information). We quantified the hydrogel stiffness before and after crosslinking (at 30, 60, 120, and 600 s) to characterize the dynamic stiffening, using shear rheology and AFM. We found that a short crosslinking time of 30 s already leads to a significant increase in matrix stiffness from ≈100 to ≈180 Pa, while a long crosslinking time of 600 s leads to a fourfold increase in matrix stiffness (Figure 2B; and Figure S2C,D, Supporting Information). Previous studies showed that an increase in Collagen I crosslinking could modulate collagen fiber density and structure.^[^ ^44^ ^]^ Therefore, we analyzed the fiber structure using reflective confocal imaging. We found that the fiber density is increased substantially in hydrogels crosslinked for 600 s (Figure 2C). Quantification of the fiber structure revealed a significant increase in fiber diameter and length and area coverage in hydrogels crosslinked for 600 s. However, shorter crosslinking times of 30, 60, and 120 s showed no significant changes in the fiber diameter, length, and density (Figure 2D–F; and Figure S2E,F, Supporting Information). Scanning electron microscopy (SEM) images supported the findings of the reflective confocal imaging (Figure S2G, Supporting Information).

As a crosslinking time of 120 s results in a minimal increase of stiffness compared with 60 s (see Figure 2B), we used the 30 and 60 s crosslinking times that correspond to ≈180 and ≈240 Pa stiffness for our cellular studies. Using this approach, we generated a robust hydrogel system that allows vascular networks to form in a fibrous matrix capable of dynamically and controllable stiffening without modulating fibrous structure or cellular viability.

### 2.3 Hydrogel Stiffening Modulates Vascular Phenotype and Adherens Junction Integrity

**Figure 3:**
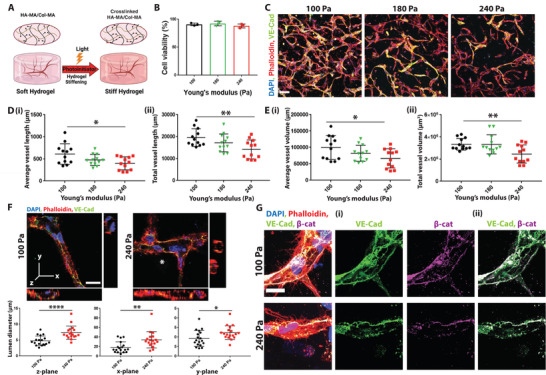
Dynamically stiffening matrix impacts microvascular phenotype and adherens junctions. A) Schematic of the 3D hydrogel system. iECs embedded in the Col‐HA hydrogel are allowed to form networks for 48 h, after which gels were crosslinked for various amounts of time. Stiffened hydrogels with networks were incubated for another 24 h before analysis. After the vascular network was established, stiffness was gradually increased to mimic the natural process of aging. B) Viability assay of iECs 24 h after stiffening (180 and 240 Pa) compared to soft hydrogels (100 Pa) (*N* = 3 biological replicates). C) Representative images of maximum intensity projection of confocal z‐stack of capillaries in soft hydrogels (100 Pa) and stiffened hydrogels (180 and 240 Pa) show that increase in stiffness leads to a decrease in Di) average and ii) total vessel length and Ei) average and ii) total vessel volume. DAPI in blue, phalloidin in red, and VE‐Cad in green. The scale bar is 100 µm (*N* = 4 biological replicates with 3 images analyzed per sample). F) Representative orthogonal images of vessels 24 h after stiffness increase in soft hydrogels (100 Pa) and stiffened hydrogels (240 Pa); the corresponding quantification of lumen diameter in each plane below the images. DAPI in blue, phalloidin in red, and VE‐Cad in green; scale bar is 20 µm (a total of 18 vessels from 3 biological replicates were analyzed). G) Representative confocal images of adherens junctions in soft hydrogels (100 Pa) and stiffened hydrogels (240 Pa) show dissociation of *β*‐catenin with increasing stiffness: i) VE‐Cad in green, DAPI in blue, phalloidin in red, and *β*‐catenin in magenta. ii) colocalization is shown using an overlay of the VE‐Cad and *β*‐catenin channels. Scale bar is 20 µm. Significance levels were set at *p* > 0.05, *p ≤ 0.05, **p ≤ 0.01, ***p ≤ 0.001, and ****p ≤ 0.0001.

After establishing and quantifying the fibrous structure of the hydrogel system, we encapsulated iECs in the hydrogels (≈100 Pa) and allowed the formation of microvascular networks for 48 h. After establishing vascular networks, the gels were photo‐crosslinked to increase the matrix stiffness (**Figure** **3**A). We first determined that increasing stiffness up to a Young's modulus of 240 Pa does not influence cell viability (Figure 3B). Thus, it is safe to test how dynamic stiffening affects 3D vascular networks. Interestingly, we found that network morphology changes occurred after moderate increases in stiffness (to 180 Pa), with significant changes in vessels subjected to a stiffness increase to 240 Pa (Figure 3C; and Figure S3A, Supporting Information). An increase in matrix stiffness leads to a decrease in vessel length, volume, and density (Figure 3D,E). Although the overall vessel volume is decreasing, most likely due to a reduction in length, we observed a general increase in lumen size with increased ECM stiffness (Figure 3F; and Figure S3B, Supporting Information). Previous work showed that adherens junction integrity is strongly reduced when ECs were cultured on stiff substrates.^[^ ^35^ ^]^ It also has been demonstrated that catenin such as *β*‐, *α*‐, and *γ*‐catenin are associated with VE‐Cadherin, stabilizing the adherens junction while connecting it to the cytoskeleton. Dissociation of *β*‐catenin upon phosphorylation of VE‐Cad leads to destabilization and disassembly of adherens junctions.^[^ ^45^ ^]^ Indeed, we observed that upon an increase in matrix stiffness, the integrity of VE‐Cad‐mediated adherens junctions is diminished, shown by the dissociation of *β*‐catenin from membrane‐associated VE‐Cad (Figure 3G).

### 2.4 An Increase in Cell Contractility Mediated Strain Stiffening Promotes Vascular Dysfunction Through Adherens Junction Disassembly

Various cell types such as fibroblasts and mesenchymal stem cells have been shown to alter their extracellular microenvironment, including collagen fiber structure near the cell surface, thereby changing the mechanical properties in their immediate microenvironment and subsequently cell signaling and cell fate.^[^ ^41^, ^46^, ^47^ ^]^ It has been suggested that cell contractility plays a significant role in mediating strain stiffening. In this scenario, actin‐mediated cell contraction leads to strain‐mediated deformation of collagen fibers, thereby increasing ECM stiffness.^[^ ^46^ ^]^ Therefore, contractile cells can induce a long‐range stiffness gradient in an enzyme‐independent manner, altering the mechanical properties near the cell surface with consequences on mechano‐sensitive cell signaling pathways.^[^ ^46^, ^48^ ^]^ Additionally, it has been shown that ECs mediate ECM stiffening during vasculogenesis.^[^ ^41^ ^]^

**Figure 4:**
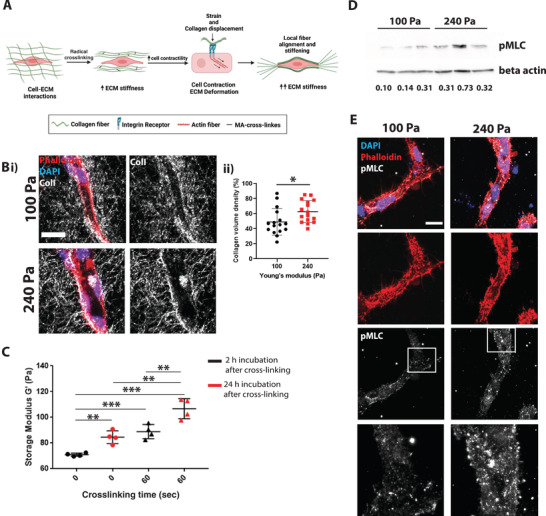
Increase in collagen crosslinking increases cell contractility and stress stiffening. A) Schematic of cell contractility mediated stress stiffening. Bi) Reflective confocal images of vessels embedded in hydrogel show an increase in collagen fiber density near the cell surface. Collagen is in gray, DAPI in blue, and phalloidin in red. Scale bar is 20 µm. ii) quantification of collagen density shows increased collagen volume with increasing stiffness near the cells. *N* = 16 vessels analyzed from 3 biological triplicates. C) Rheology measurements 2 and 24 h after crosslinking show a significant increase in stiffness after crosslinking compared to control. It further shows a continuous increase in stiffness along culturing time. D) Western blot shows increased levels of pMLC in ECs subjected to stiffness increase 24 h after crosslinking. pMLC levels were normalized to beta‐actin are shown below the bands. E) pMLC staining of ECs show increased pMLC levels with increasing stiffness (DAPI in blue; phalloidin in red; pMLC in gray). Images to the right are 3X amplification of the boxed area. The scale bar is 20 µm. Significance levels were set at *p* > 0.05, *p ≤ 0.05, **p ≤ 0.01, ***p ≤ 0.001

Here we hypothesized that this effect of strain stiffening might further impact the stiffness of the hydrogel, thus adding to the exogenous chemically induced stiffening (**Figure** **4**A). Therefore, we measured collagen density and structure near the cells using transmission electron microscopy (TEM) and reflection confocal imaging. We found higher collagen density near iECs in hydrogels that underwent stiffening than soft hydrogels (Figure 4B; and Figure S4A,B, Supporting Information). Further, TEM and reflection microscopy showed an evident change in collagen structure near the cell surface, with longer, more aligned collagen fibers in stiffened matrices (Figure 4B; and Figure S4A, Supporting Information). We next sought to measure the bulk stiffness of hydrogels that underwent stiffening to verify the increase in stiffness due to collagen remodeling over time. We embedded iECs into hydrogels and let them form a vascular network for 48 h. After network formation, the hydrogels were left untreated or photo‐crosslinked for 60 s, and stiffness was measured 2 or 24 h after cross‐linking. We found a continuous increase in stiffness even 24 h post‐crosslinking (Figure 4C). Because strain stiffening is dependent on cell contractility, we next examined the phosphorylation of the nonmuscular myosin light chain (MCL). We found that pMLC is upregulated in the stiffening matrix (Figure 4D,E; and Figure S4C,D, Supporting Information). An increase in collagen deposition is well characterized in large blood vessels, mainly the aorta.^[^ ^49^ ^]^ Upon aging, a loss of elastin and increased collagen deposition and crosslinking have been observed.^[^ ^50^, ^51^ ^]^ Because collagen deposition and degradation are strongly dependent on Matrix Metalloproteinases (MMP) activity, we examined the change in expression of the membrane‐type 1 MMP (MT1‐MMP), which directly degrades the ECM and activates soluble MMPs. We could not detect a difference in vascular network expression of MT1‐MMP between the untreated and stiffening matrix (Figure S4E, Supporting Information).

### 2.5 Inhibition of Cell Contractility Rescues Stiffness Induced Vascular Morphology and Restores Adherens Junctions

Cell contractility is an important regulator of EC migration and vascular network formation. Inhibition of MLC phosphorylation was previously shown to reduce endothelial sprouting and subsequent vessel formation.^[^ ^36^, ^52^ ^]^ Further, the inhibition of cell contractility was shown to reduce adherens junctions and barrier function by promoting vessel dilation and disorganization of VE‐Cad at the cell–cell junction.^[^ ^52^, ^53^ ^]^ However, other research showed that an increase in pMLC levels in ECs, most likely in a Rho/Rock dependent manner, further destabilizes adherens junctions and subsequently decreases barrier function.^[^ ^54^, ^55^ ^]^These somewhat contrary findings indicate that cell contractility can have an opposite role in maintaining adherens junction stability ranging from stabilizing to destabilizing.

**Figure 5:**
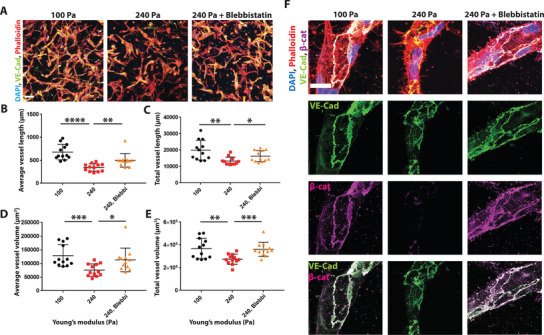
Inhibition of MLC phosphorylation with blebbistatin rescues stiffening induced phenotype. A) Representative confocal images of maximum Intensity projection of stiffened hydrogels in the presence of blebbistatin show that blebbistatin rescues stiffness mediated decrease in B,C) vessel length and D,E). vessel volume. DAPI in blue, phalloidin in red, VE‐Cad in green. Scale bar 100 µm. (*N* = 3 biological replicates with 4 images analyzed per sample). F) Representative confocal images show rescue of adherens junctions in blebbistatin treated vessels, VE‐Cad in green, *β*‐catenin in magenta, DAPI in blue, and phalloidin in red. Far right images show an overlay of the VE‐Cad and *β*‐catenin channel. Scale bar is 20 µm. Significance levels were set at *p* > 0.05, *p ≤ 0.05, **p ≤ 0.01, ***p ≤ 0.001, and ****p ≤ 0.0001.

To study whether cell contractility contributes to the observed capillary phenotype, we treated the vessels in the stiffened hydrogels with a cell contractility inhibitor, blebbistatin. We allowed the iECs to form capillary networks for 48 h, followed by stiffening via crosslinking. Directly after crosslinking, we added the blebbistatin (final concentration of 6 × 10^−6^ m) to the culture media and compared vascular morphology to untreated stiffened conditions and vehicle control. Treatment of the vascular network with blebbistatin rescues the stiffening‐induced vascular phenotype (**Figure** **5**A), with a significant increase in vessel length and volume (Figure 5B–E). Further, blebbistatin treatment did not only rescue the stiffening‐induced vessel shortening and volume reduction but also rescued the loss of adherens junctions, with a noticeable increase in membrane localization of VE‐Cad and *β*‐catenin (Figure 5F). Interestingly, when treating with blebbistatin, vascular networks that were not subjected to crosslinking (100 Pa hydrogels), we observed the opposite phenotype with reduced vascular length and reduced VE‐Cad localization at the cell membrane (Figure S5A–C, Supporting Information). Together, our data show that cell contractility is well balanced in ECs, and a shift toward either upregulation or downregulation has compromised endothelial barrier function. Further, we show that correcting this balance upon an increase in matrix stiffness leads to a recovery of the vascular morphology.

### 2.6 Cell Contractility and Increased pMLC Levels Regulate Vascular Morphology via FAK Phosphorylation and Activation

**Figure 6:**
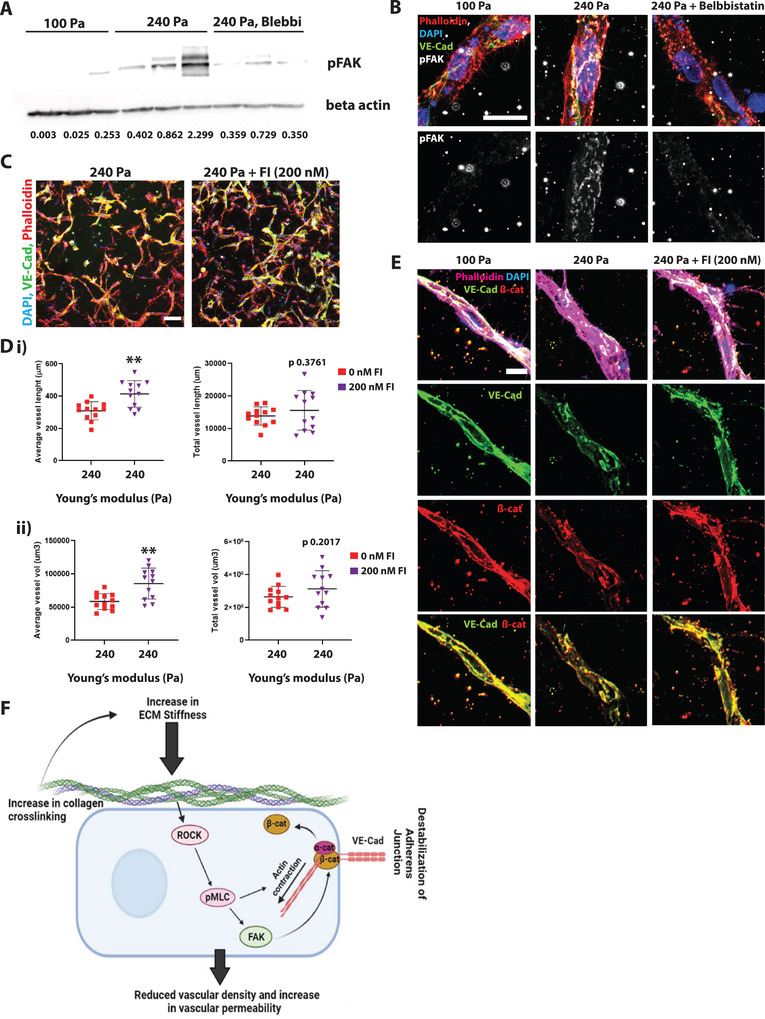
Inhibition of cell contractility reduces pFAK levels, thus recovering the vascular phenotype: A) Western blot and quantifications for pFAK levels in the soft (100 Pa) and stiffened (240 Pa) hydrogels. pFAK levels were normalized to beta‐actin are shown below the bands. B) Representative confocal images of maximum projection 24 h post‐crosslinking. pFAK levels are increased in samples subjected to stiffness increase, as shown by larger pFAK clusters (arrows). Treatment with Blebbistatin reduces pFAK levels (DAPI in blue, phalloidin in red, VE‐Cad in green, and pFAK in gray). The scale bar is 20 µm. C) Representative confocal images of maximum Intensity projection of stiffened hydrogels in the presence of 200 × 10^−9^ m FAK inhibitor show rescue on D,i) vessel length and D,ii) vessel volume. E) Representative confocal images show rescue of adherens junctions in FAK inhibitor (200 × 10^−9^ m) treated vessels (VE‐Cad in green, *β*‐catenin in red, DAPI in blue, and phalloidin in magenta). The bottom images show an overlay of the VE‐Cad and *β*‐catenin channels. Scale bar is 20 µm. Significance levels were set at *p* > 0.05, *p ≤ 0.05, **p ≤ 0.01. F) Schematic of the suggested pathway showing that pMLC regulates vascular phenotype in a FAK‐dependent manner in stiffening matrix.

Focal adhesion kinase (FAK) activity strongly correlates with EC contractility.^[^ ^36^ ^]^ Thus, we examined whether pMLC increase correlates with FAK activity in the stiffening hydrogels. We found that FAK activity has increased 4 h poststiffening, as shown by an increase in FAK phosphorylation (**Figure** **6**A). In the next step, we examined whether inhibition of cell contractility reduces FAK activity. The capillary networks were treated with blebbistatin directly after crosslinking, followed by an analysis of pFAK at 4 h poststiffening. Indeed, we found that inhibition of cell contractility using Blebbistatin leads to a decrease in phosphorylated FAK within 4 h after photo‐crosslinking (Figure 6A). Blebbistatin treatment further reduced pFAK expression 24 h after matrix stiffening (Figure 6B). To investigate whether cell contractility regulates vascular morphology via activation of FAK, we treated the vessels with FAK inhibitor (PF‐573228) after photo‐crosslinking. When cultured in 2D, FAK inhibitor concentrations between 200 and 800 × 10^−9^ m modulated endothelial adherens junctions (Figure S6A, Supporting Information). Indeed, treatment with 200 × 10^−9^ m of FAK inhibitor led to a rescue in average vessel length and volume following matrix stiffening (Figure 6C,D). Examining the adherens junctions showed increased VE‐Cad membrane expression and colocalization of *β*‐cat with VE‐Cad at the cell membrane in vessels treated with 200 and 800 × 10^−9^ m of FAK inhibitor (Figure 6E). Thus, we conclude that cell contractility regulates vascular morphology and adherensjunction integrity via the activation of FAK and that inhibition of cell contractility leads to a rescue of matrix stiffening induced vascular morphology (Figure 6F).

## 3 Conclusion

In our work, we show a 3D in vitro hydrogel system capable of increasing its stiffness during cell culture, thus mimicking the process of microvascular aging and unraveling age‐mediated changes in cell signaling. The effect of aging on capillary morphology and function has been previously addressed, and it is known that aging has a dramatic impact on capillary density and function.^[^ ^12^ ^]^ However, the underlying cell signaling events leading to age‐mediated capillary dysfunction are not well characterized. So far, it is generally assumed that the reduction in capillary density upon aging is due to reduced angiogenesis.^[^ ^56^ ^]^ A recent study by Chen et al. revealed that pericyte to fibroblast differentiation in aging organs might be another reason for reduced capillaries in aged organs.^[^ ^12^ ^]^ An established hallmark of aging is a change in the mechanical properties of the tissue ECM such as an increase in stiffness.^[^ ^57^ ^]^ In this study, we utilized a 3D hydrogel system to mimic the increase in ECM stiffness via increasing collagen crosslinking. To do so, we utilized Col‐MA and HA‐MA to generate a physiological fibrillar matrix. We increased crosslinking by using a photoinitiator and visible light to protect the cells from UV induced damage. We found that the stiffening matrix promotes an increase in cell contractility leading to an increase in FAK activation resulting in the dissociation of *β*‐catenin from VE‐Cad and the adherens junction destabilization. Our data further show that cell contractility is well balanced in ECs, and shifts to either side can have detrimental effects on adherens junction stability. Thus, maintaining this balance can positively impact vessel stability and adherens junction with increased stiffness and help maintain a healthy capillary phenotype. Previous studies found that cell contractility is necessary during angiogenesis and maintaining adherens junction integrity;^[^ ^36^, ^52^, ^53^ ^]^ however, other studies highlighted an adverse effect of cell contractility on adherens junction integrity.^[^ ^54^, ^55^ ^]^ Our findings thus further support the hypothesis that well‐balanced cell contractility and pMLC levels are needed, with detrimental effects if the balance is tipped to either side.

Although cell contractility plays a major role in controlling vascular morphology, we could not see a direct correlation between the observed increase in vessel lumen diameter and cell contractility. However, vascular aging is a complex process involving multiple cell signaling events. Thus, further investigation into age‐mediated EC signaling is necessary. Nonetheless, our system recapitulates and correlates with observations of reduced capillary density in various organs with increasing age.^[^ ^12^ ^]^ Indeed, we found that aging leads to an increase in overall tissue stiffness, collagen deposition, and reduced capillary density in the kidneys of aged mice. Our studies focused on the increase in collagencrosslinking and subsequent increase in stiffness and its effect on endothelial signaling. However, given that changes in ECM composition have been observed during the process of aging, future studies should examine how ECM molecules such as collagens, fibronectin, and laminin can further influence cell signaling.^[^ ^58^, ^59^, ^60^ ^]^Moreover, aging is a complex process involving inflammation,^[^ ^61^ ^]^ oxidative stress,^[^ ^62^ ^]^ and DNA damage.^[^ ^63^ ^]^ Thus, future research involving more advanced biomaterials and culture systems can mimic different aspects of aging. Our approach is the first to study a dynamic increase in tissue stiffness after forming a vascular network, demonstrating the effect on cellular contractility and vascular network phenotype, with implications for basic and translational studies.

## 4 Experimental Section

### Cell Culture and iPSC Differentiation

Undifferentiated C1‐2 induced pluripotent stem cells^[^ ^64^ ^]^ were maintained on vitronectin (Thermo Fisher Scientific) coated plates and cultured in Essential 8 media (Thermo Fisher Scientific).^[^ ^65^, ^66^ ^]^ Human iPSCs were differentiated to endothelial cells (iECs) as described previously.^[^ ^65^, ^66^, ^67^ ^]^ In brief, differentiation was induced on undifferentiated cells cultured to 80% confluency with Essential 6 medium (Thermo Fisher Scientific) supplemented with 6 × 10^−6^ m CHIR (STEMCELL Technologies) for 48 h with media changed daily. After 48 h, cells were digested in TrypLE Express (Thermo Fisher Scientific) and seeded on collagen type I‐coated plates at a density of 2 × 10^4^ cells cm^−2^ in Endothelial Cell Growth Medium (ECGM; Promocell) supplemented with 10 × 10^−6^ m SB‐431542 (Cayman Chemical Company) and 50 ng mL^−1^ vascular endotheial growth factor (VEGF; R&D Systems), with additional supplementation of 10 × 10^−6^ m Y‐27632 for the first 24 h. After the first 24 h, the media was changed every other day for an additional 6 days.

### Isolation of iECs and Expansion

iECs were isolated and expanded as described previously.^[^ ^66^, ^67^ ^]^ In brief, CD31‐expressing cells were isolated via magnetic‐activated cell sorting (MACS; Miltenyi Biotec Bergisch Gladbach), following the manufacturer's protocol, on day 8 of differentiation. After rinsing the cells with 1x phosphate‐buffered saline (PBS; Thermo Fisher Scientific), cells were trypsinized with TrypLE Express and resuspended in MACS buffer (0.5 Ethylenediaminetetraacetic acid [MilliporeSigma] and 0.5% Bovine Serum Albumin (BSA; [MilliporeSigma] in PBS). After resuspension, cells were incubated with 10 µL of PE‐conjugated anti‐human CD31 (BD Biosciences) for 10 min at 4 °C. To remove the unbound primary antibody, cells were washed twice with MACS buffer. After washing, cells were resuspended in 80 µL MACS buffer, and 20 µL of anti‐PE microbeads (Miltenyi Biotec Bergisch Gladbach) were added to the cell suspension. Cells were incubated for an additional 15 min at 4 °C, followed by a washing step with MACS buffer before separation using the MS MACS separation column (Miltenyi Biotec Bergisch Gladbach). Finally, CD31^+^ cells were seeded on collagen type I‐coated plates and maintained in ECGM supplemented with 50 ng mL^−1^ VEGF and 10 × 10^−6^ m SB‐431542.

### Hydrogel Preparation and Characterization

To synthesize HA‐MA, 1.0 g of sodium salt of HA was dissolved in 50 mL of deionized water. 1.2 mL Methacrylic anhydride was added dropwise while stirring at 4 °C. The stirring mixture was maintained at pH 8–8.5 by continuously adding 1 m NaOH solution for ≈8 h. The mixture was dialyzed (MWCO 8000) against NaCl solution and DI water for 3 d, respectively, then frozen at −80 °C and subsequently lyophilized to powder form. The degree of methacrylate modification was determined as 36% from 1H NMR spectra by integrating peaks at 3.2–4.3 ppm and peaks at 5.8 ppm, and peaks at 6.4 ppm, which corresponded to methyacrylamides and the sugar ring of hyaluronic acid, respectively. 1H NMR spectra were recorded in D_2_O on a Bruker Advance 400 MHz Spectrometer.

For the preparation of 1 mL of HA‐MA/collagen‐MA solution, 410 µL of high VEGF media, 205 µL of Medium 199 (1x), 40 µL of Medium 199 (10x), 312.5 µL of bovine Collagen‐MA (final concentration 2.5 mg mL^−1^, Advanced Biomatrix, 5189), and 27.5 µL HA‐MA (final concentration 0.25 mg mL^−1^) were mixed on ice. The hydrogel solution was then neutralized using neutralization solution (Advanced Biomatrix, 5205), and hydrogels were allowed to polymerize at 37 °C for 30 min. After gelation, photoinitiator was prepared according to the manufacturer's protocol (Advanced Biomatrix, 5248). In brief, Ruthenium (Ru) (37.4 mg mL^−1^) and Sodium Persulfate (SPS) (119 mg mL^−1^) were diluted in high VEGF medium to a final concentration of 0.7% w/v Ru and 2% w/v SPS. Photo‐crosslinker solution was added on top of the gels and incubated for 10 min to allow the crosslinker to diffuse into the gels. For the stiffening experiments, hydrogels underwent secondary crosslinking using visible light for the times indicated in the manuscript.

### Hydrogel Stiffness Measurements

Bulk stiffness was measured using an AR‐G2 Rheometer (TA Instruments) equipped with an 8 mm parallel plate at 37 °C as previously.^[^ ^36^ ^]^ Storage modulus *G*' was monitored at a fixed strain rate of 1% and fixed frequency of 1 Hz. All hydrogels were prepared as discs measuring 8 mm in diameter.

Atomic force microscopy (AFM) experiments were performed with a Silicon Nitride (SiN) cantilever (nominal spring constant of 0.06 N m^−1^) with spherical SiO_2_ tips with a diameter of 5 µm. (PT.GS, Novascan, USA) on an MFP3D (Asylum Research, USA) instrument. The thermal fluctuation method was used to calibrate the stiffness of the cantilever before every experiment.^[^ ^68^ ^]^ To measure the stiffness (Young's modulus) of the hydrogel samples, force‐displacement curves were obtained using contact mode. Indentation data were processed using Igor‐pro software (Wavemetrics, USA). Young's modulus was obtained by fitting the force‐displacement curves with the Hertz model, which related the applied force (F) by the cantilever tip to the indentation (*δ*) and Young's modulus (E) using the following equation

(1) $$F=\frac{2E\tan a}{\pi \left(1-\nu^{2}\right)}\sqrt{R}\delta^{3/2}$$

Where *α* is the tip opening angle (35°), *R* is the radius of the spherical indenter, and *v* is the Poisson ratio (which is assumed to be 0.5 for soft biological materials.^[^ ^69^ ^]^

### Reflective Confocal Imaging of Collagen Fibers and Fiber Analysis

Reflective microscopy was used to analyze collagen fibers in the Col‐HA gels. Images were collected using an LSM 780 (Zeiss) microscope. A 40x oil immersion objective and 561 nm light were used for reflective microscopy to illuminate and capture collagen fibers. Collagen fiber diameter, length, and coverage area were analyzed using a previously developed method based on MATLAB (Mathworks) software MatFiber.^[^ ^70^ ^]^ 21 images from 3 independent replicates were analyzed, and average fiber length and diameter and a probability distribution were plotted using GraphPad Prism.

### Collagen Fiber Analysis

Vessel formation was allowed for 48 h, and hydrogel stiffness was increased, as detailed above. Hydrogels were fixed 48 h poststiffening, and vessels were visualized using phalloidin. Collagen fibers were visualized using reflective microscopy. Images were collected using an LSM 780 (Zeiss) microscope. Region of interests (ROI) (44 × 49 × 12 µm^3^) were created around vessels. Total collagen area and total vessel area were calculated using ImageJ software. Collagen fiber density was calculated as the ratio of collagen volume to the difference of total ROI to vessel volume.

### Cell Encapsulation and Hydrogel Crosslinking

For iECs encapsulation in 1 mL of Col‐MA/HA‐MA solution, 8 × 10^5^ cells were resuspended in 205 µL ECGM supplemented with 50 ng mL^−1^ VEGF (high VEGF media). Before adding the cell suspension, the hydrogel solution was prepared and neutralized as described above. Finally, 60 µL of hydrogel solution were plated in a 96 well‐plate precoated with Collagen I. The hydrogels were polymerized for 30 min at 37 °C. After gelation, 200 µL of high VEGF media were added to the 3D hydrogels and cultured at 37 °C, 5% CO_2_ for 2 days to allow vasculogenesis. After the capillary network was established, the constructs were subjected to cross‐linking. Photoinitiator was prepared, added to the constructs, and incubated as detailed above. After crosslinking, the constructs were extensively washed with high VEGF media containing 0.2% dimethyl sulfoxide (DMSO) and cultured in high VEGF media containing 0.2% DMSO for another 24 h before analysis.

### Cell Viability

iECs were encapsulated into hydrogels as described above. To assess the proper incubation time for the photoinitiator, the gels were incubated with a photoinitiator for 5, 10, and 30 min. After incubation, gels were washed using endothelial cell growth media and incubated in EC diff media for another 24 h. Cell viability was assessed using calcein‐AM and ethidium homodimer‐1 (Live/Dead kit, Thermo Fisher, L3224).

To determine cell viability for stiffness experiments, iECs were encapsulated into hydrogels, allowing network formation for 48 h. The networks were then incubated with photoinitiator solution for 10 min and crosslinked for the indicated time. After crosslinking, the constructs were extensively washed with high VEGF media containing 0.2% DMSO and cultured in high VEGF media containing 0.2% DMSO for another 24 h. Cell viability was assessed using calcein‐AM and ethidium homodimer‐1 (Live/Dead kit, Thermo Fisher, L3224) and LSM780 confocal microscope (Zeiss) and analyzed using ImageJ software. ^[^ ^71^ ^]^

### Immunofluorescence Staining, Imaging, and Network Analysis

Hydrogels containing capillary networks were fixed using 4% Paraformaldehyde (PFA, Sigma‐Aldrich) for 20 min at room temperature. The constructs were then washed 3 times with PBS to remove the PFA thoroughly. After fixation, constructs were permeabilized with 1% Triton‐X (Sigma‐Aldrich) in PBS for 30 min at room temperature. The constructs were then incubated with primary antibody diluted (1:100) in 1% Triton‐X in PBS overnight at 4 °C. After that, the hydrogels were washed 3 times 15 min with 1% Triton‐X in PBS and incubated for 2 h at room temperature in the dark with a secondary antibody diluted (1:500) in 1% Triton‐X in PBS. After the incubation with secondary antibody, the constructs were counterstained with DAPI (1:1000, Thermo Fisher Scientific) for 15 min at room temperature in the dark. After DAPI staining, the constructs were washed 3 times for 15 min with 1% Triton‐X in PBS and imaged using LSM 780 or LSM 800 confocal microscopy (Zeiss). Primary antibodies include mouse anti‐VE‐Cad (Santa Cruz, sc‐9989), rabbit anti‐*β*‐catenin (Sant Cruz, sc‐7199), rabbit anti‐pMLC (Cell Signaling, 3678S), rabbit anti‐pFAK (Thermo Fisher Scientific, 44624G), and MT‐1‐MMP (Abcam, ab51074) were diluted 1:100 in 1% Triton‐X in PBS. Secondary antibodies include Alexa Fluor 488 Goat anti‐Mouse IgG (Thermo Fisher Scientific, A11029), Alexa Fluor 635 goat anti‐rabbit IgG (Thermo Fisher Scientific, A31577), and Alexa Fluor 546 phalloidin (Thermo Fisher Scientific, A22283) were diluted 1:500 in 1% Triton‐X PBS.

Constructs were imaged using LSM780 and LSM800 confocal microscope (Zeiss) and visualized using ImageJ software. ^[^ ^71^ ^]^ For network analysis, z stacks of 200 µm were taken and analyzed using the Imaris Filament tracer (Imaris version 9.0, Bitplane).

### TEM

Samples were fixed in 4% glutaraldehyde, 5 × 10^−3^ m CaCl_2,_ 5 × 10^−3^ m MgCl_2_, 0.1% tannic acid in 0.1 m sodium cacodylate buffer, pH 7.2 overnight at room temperature. After buffer rinse, samples were postfixed in 1% osmium tetroxide reduced with 0.8% potassium ferrocyanide in 0.1 m sodium cacodylate (1 h) on ice in the dark. Following a dH_2_O rinse, samples were en bloc stained with 2% uranyl acetate (aq.) followed by dehydration in a graded series of ethanol and embedded in Eponate 12 (Ted Pella) resin. Samples were polymerized at 60 °C overnight.

Thin sections, 60–90 nm, were cut with a diamond knife on a Leica Ultracut UCT ultramicrotome and picked up with 2 × 1 mm formvar coated copper slot grids. Grids were triple stained with 1% tannic acid and 2% uranyl acetate, followed by 0.4% lead citrate and imaged on a Talos L120C at 120 kV. Images were captured with a Thermo‐Fisher Ceta (cooled 16 Mpixel CMOS, 16‐bit 1–25 fps).

### SEM

Samples were dehydrated through a graded series of ethanol; critical point dried using a LADD CPD3 critical point dryer and coated with 7 mm gold using a Denton Desk V sputter coater. Samples were imaged in an Apreo S by ThermoFisher Scientific using the Everhart–Thornley secondary electron detector at 2.0 keV under a high vacuum.

### Western Blotting

Hydrogels were lysed on ice using RIPA buffer (Thermo Fisher Scientific). After constructs were lysed, sodium dodecyl sulfate (SDS) loading buffer (4X laemmli sample buffer, BioRad Ref.1610747) with 1x sample reducing agent (Thermo Fisher, B0009) was added. The protein solution was boiled at 95 °C for 5 min and then loaded onto a 10% SDS Gel. Protein was transferred to a polyvinylidene fluoride membrane (Bio‐Rad) via wet transfer (Bio‐Rad Criterion system). The membrane was blocked with 3% BSA in TBST (20 × 10^−3^ m Tris, pH 7.4, 150 × 10^−3^ m NaCl, 0.1% Tween‐20) for 40 min and probed with primary antibody overnight at 4 °C. Membranes were washed with TBST and probed with secondary IgG HPR‐conjugated antibody (Cell signaling Technologies) for 90 min at room temperature. The membranes were rewashed with TBST, probed with Clarity Western ECL Substrate (Bio‐Rad), and imaged using the ChimiDoc XRS+ System (Bio‐Rad). Blots were analyzed using ImageJ, and bands were normalized to *β*‐actin expression.

### Tissue Stiffness Measurements

All animal procedures complied with the NIH Guidelines for the Care and Use of Laboratory Animals and were approved by the Institutional Animal Care and Use Committee. Kidneys were measured using an AR‐G2 Rheometer (TA Instruments) equipped with an 8 mm parallel plate at room temperature. Storage modulus *G'* was monitored at a fixed strain rate of 1% and fixed frequency of 2 Hz. For tissue AFM, the same method of indentation and analysis was used as described for the hydrogels. Cryosections of 25 µm in thickness were stained for collagen using ColF, and cells were visualized by nuclear Hoechst staining. Indentations were performed at various locations highlighted by collagen staining (ColF). Additionally, it was ensured the analyzed locations had a low density of cells as identified using Hoescht Stain. Multiple indentations over various locations per sample were used to get the distribution.

### Tissue Clearing

Kidneys of aged (18 + months) and young (7–9 weeks) C57/BL6 female mice were harvested following IACUC guidelines. After extraction, the kidneys were fixed and incubated in 4% PFA overnight at room temperature and stored in 70% EtOH at 4 °C until further use. For tissue clearing, the kidneys were washed with PBS for 2 h at room temperature and cut into ≈2 mm thick slices. The tissue clearing was done using the CytoVista Tissue clearing kit (Thermo Fisher, V11324), following the manufacturer's protocol with slight adaptations. After incubation with blocking buffer, the sample was incubated for 24 h with isolectin B4 (1: 250) for 24 h at 37 °C, followed by another incubation step of isolectin B4 (1:100) for 48 h at 37 °C. Samples were imaged using an LSM780 confocal microscope (Zeiss) and visualized using ImageJ software. Vascular density was analyzed using Imaris Filament tracer (Imaris version 9.0, Bitplane). To calculate vascular density, the total vascular volume was normalized to the total volume of the imaged section.

### Tissue IHF

Optimal cutting temperature compound embedded frozen kidneys were sectioned and stained as previously described. In brief, 5 µm kidney cryosections of old (18 + months) and young (7–9 weeks) female C57/BL6 mice were fixed with 4% PFA at room temperature for 10 min and washed twice for 5 min with PBS at room temperature. Tissue was blocked using 1% goat serum in PBS for 1 h at room temperature before incubation with primary antibody (1:100) (anti‐Collagen I, Novus Biological) overnight at 4 °C. Samples were washed 3 times in PBS for 10 min each at room temperature and incubated with secondary antibody (1:1000) for 2 h at room temperature. Slides were mounted with and imaged using LSM800 confocal microscope (Zeiss). Images were visualized and analyzed using ImageJ software.

### Statistical Analysis

All experiments were performed in biological triplicates (*N* = 3); image‐based quantifications were performed in at least *N* = 3 biological samples and detailed throughout the methods and figure legends. The filaments of vascular networks in the hydrogels were quantified using Imaris. The statistical analysis was performed using Graph Pad 7.0, and a Two‐tailed *t*‐test was performed to determine statistical significance. All graphical data are reported as mean ±SD. Significance levels were set at *p < 0.05, **p < 0.01, ***p < 0.001, and ****p < 0.0001.

## Conflict of Interest

The authors declare no conflict of interest.

## Supporting information

Supporting Information

## Data Availability

The data that support the findings of this study are available from the corresponding author upon reasonable request.
