# Supplementary material for: Stiffening Matrix Induces Age‐Mediated Microvascular Phenotype Through Increased Cell Contractility and Destabilization of Adherens Junctions
Source: Adv Sci (Weinh). 2022 Jun 3;9(22):2201483. doi: 10.1002/advs.202201483 (PMC9353494; doi:10.1002/advs.202201483)
Supplement: Supplementary file 1 — Supporting Information [file ADVS-9-2201483-s001.pdf]

Stiffening matrix induces age-mediated microvascular phenotype through increased cell contractility and destabilization of adherens junctions

Rahel Schnellmann[1,2], Dimitris Ntekoumes[1,2] Mohammad Ikbal Choudhury[2,3], Sean Sun[2,3], Zhao Wei[1,2], Sharon Gerecht [1,2,4,5,6]*

Supplementary Figures

Supplementary Figure 1

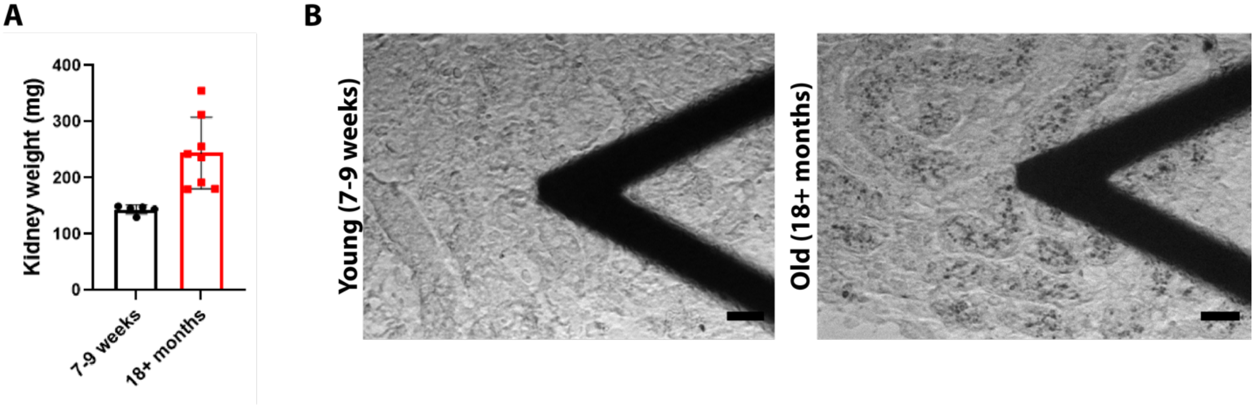

Supplementary Figure 1. Kidney size changes with age. A. Aged female mice 18+ months old have larger kidneys than young female mice (7-9 weeks). N=5 for young mice and N=8 for aged mice. **B.** Representative bright-field images of kidney slices measured by AFM. The black triangle is the cantilever.

Suplemantary Figure 2

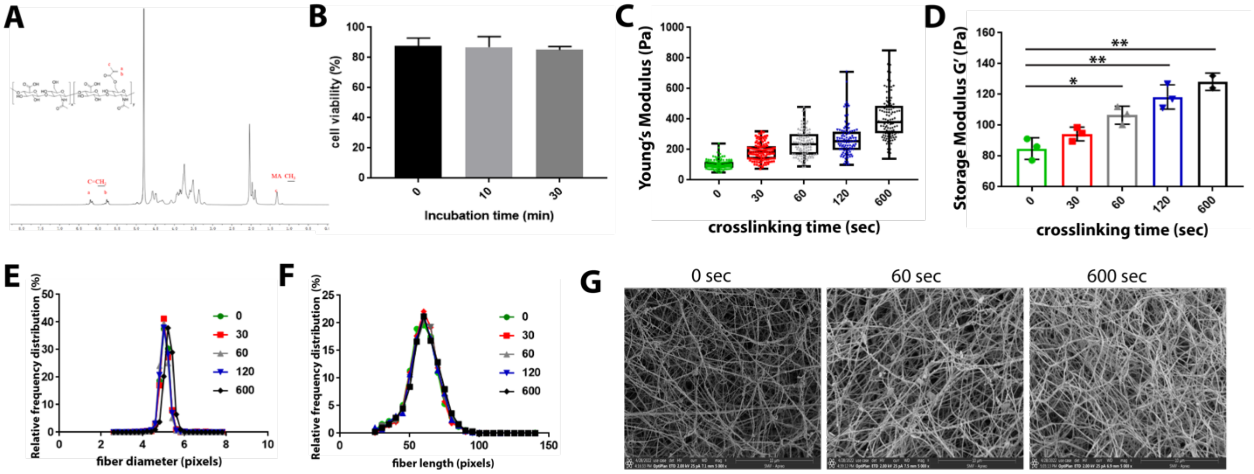

Supplementary Figure 2: Characterization of Col-MA/HA-MA hydrogels. A. Representative ^{1}H NMR spectra of HA-MA verifying successful coupling of MA. Spectra were recorded in D_2O on a 400 MHz Spectrometer. **B.** Cell viability assay of iECs incubated with ruthenium crosslinker using the calcein-AM assay. N=3 biological replicates. **C.** Details of all the collected AFM measurements and **D.** Shear rheology measurements of storage modulus of the hydrogels after different times of crosslinking. N=3 independent replicates. **E.** Fiber density- frequency distribution and **F.** fiber length-frequency distribution after different crosslinking times (N=3 with 4 images analyzed per condition). **G.** SEM images of fiber structure of hydrogels after different time points of crosslinking. Significance levels were set at $p > 0.05$, $*p \leq 0.05$, $**p \leq 0.01$, $***p \leq 0.001$ and $****p \leq 0.0001$.

Supplementary Figure 3

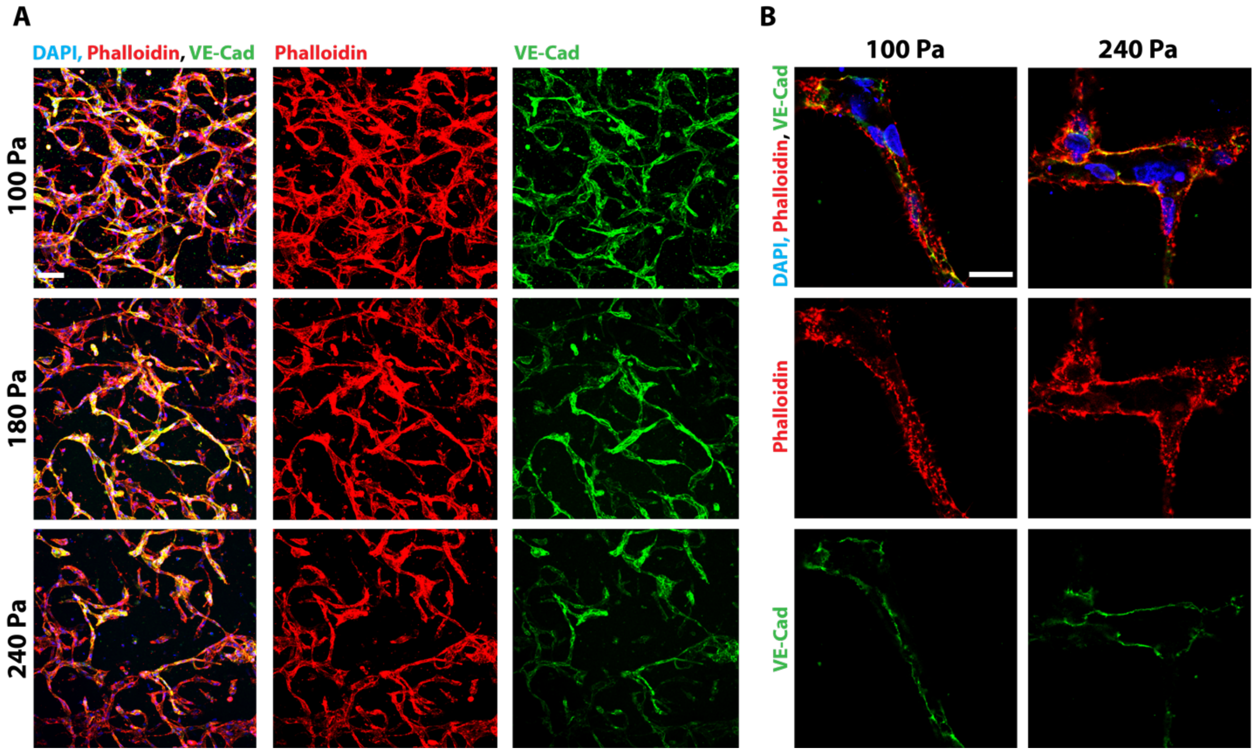

Supplementary Figure 3: Dynamically stiffening matrix affects capillary structure. A. Representative images of maximum intensity projection of confocal z-stack of capillaries in soft hydrogels (100 Pa) and stiffened hydrogels (180 Pa and 240 Pa) (phalloidin in red, DAPI in blue, and VE-Cad in green). Scale bar is 100 µm. **B.** Representative images of vessels 24 hrs after stiffness increase in soft hydrogels (100 Pa) and stiffened hydrogels (240 Pa) (phalloidin in red, DAPI in blue, and VE-Cad in green). Scale bar is 20 µm.

Supplementary Figure 4

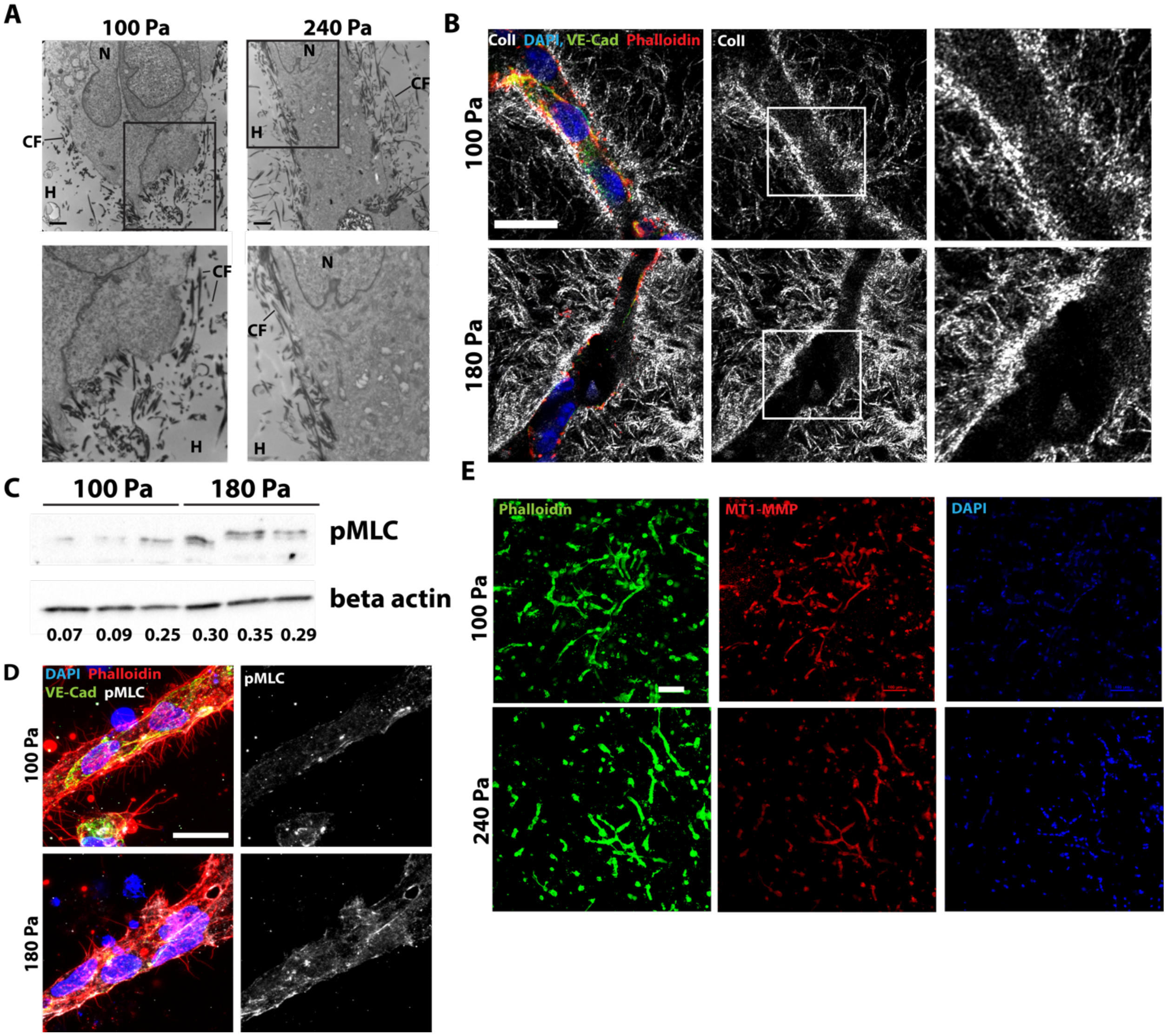

Supplemental Figure 4. Cell contractility and stress stiffening are increased in the photo-crosslinked hydrogels. A. Remodeling of the ECM near the networks after photocrosslinking. TEM images show longer and more condensed collagen fibers near the cell surface 24 hrs after cross-linking. Lower images are zoomed images of the boxed area of the image above. The scale bar is 2 μm. **B**. Reflective confocal images of vessels embedded in hydrogel show collagen fiber density near cells **C**. Western blot and quantifications for pMLC expression in the soft (100 Pa) and stiffened (180 Pa) hydrogels. **D**. pMLC staining in vessels with increasing stiffness. DAPI in blue, phalloidin in red, VE-Cad in green, and pMLC in gray. The scale bar is 20 μm. **E.** MT-1MMP staining in vessels with increasing stiffness. DAPI in blue, phalloidin in green, and MT1-MMP in red. The scale bar is 100 μm.

Supplementary Figure 5

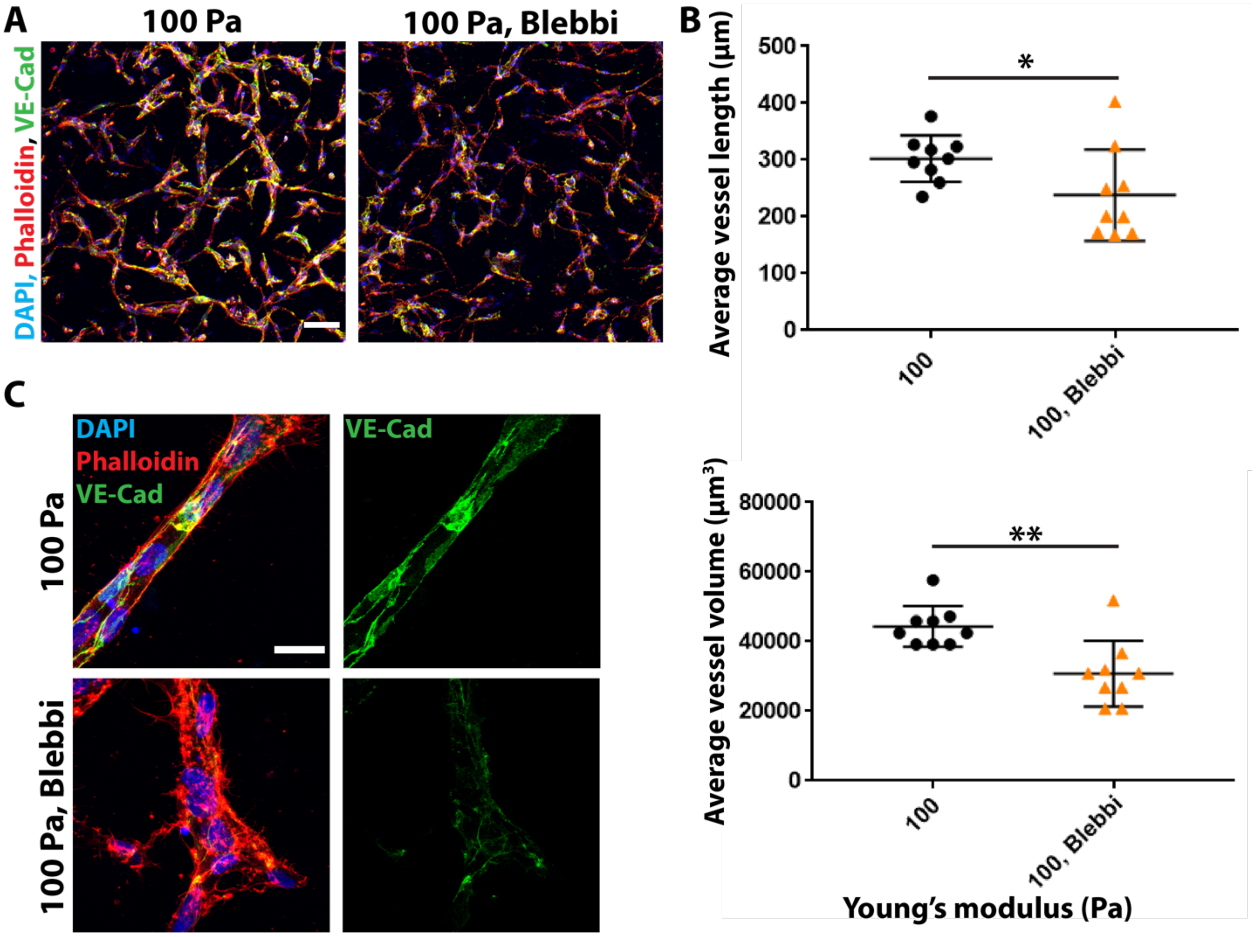

Supplementary Figure 5. Blebbistatin treatment reduces vascular length and volume and decreases VE-Cad membrane localization. A. Representative confocal images of maximum projection of vascular networks after treatment with blebbistatin. Cells were allowed to form networks for 48 hrs, after which capillary networks were treated with blebbistatin for another 24 hrs (DAPI in blue, phalloidin in red, VE-Cad in green). The scale bar is 100 μm. **B**. Quantification of vessel length (upper) and vessel volume (lower) of vessels treated with blebbistatin (N=3 biological replicates with 3 images per sample being analyzed). Significance levels were set at $p > 0.05$, $*p \leq 0.05$, $**p \leq 0.01$. **C.** Representative confocal images of maximum projection of VE-Cad in vessels treated with blebbistatin. (DAPI in blue, phalloidin in red, VE-Cad in green). The scale bar is 20 μm.

Supplementary Figure 6

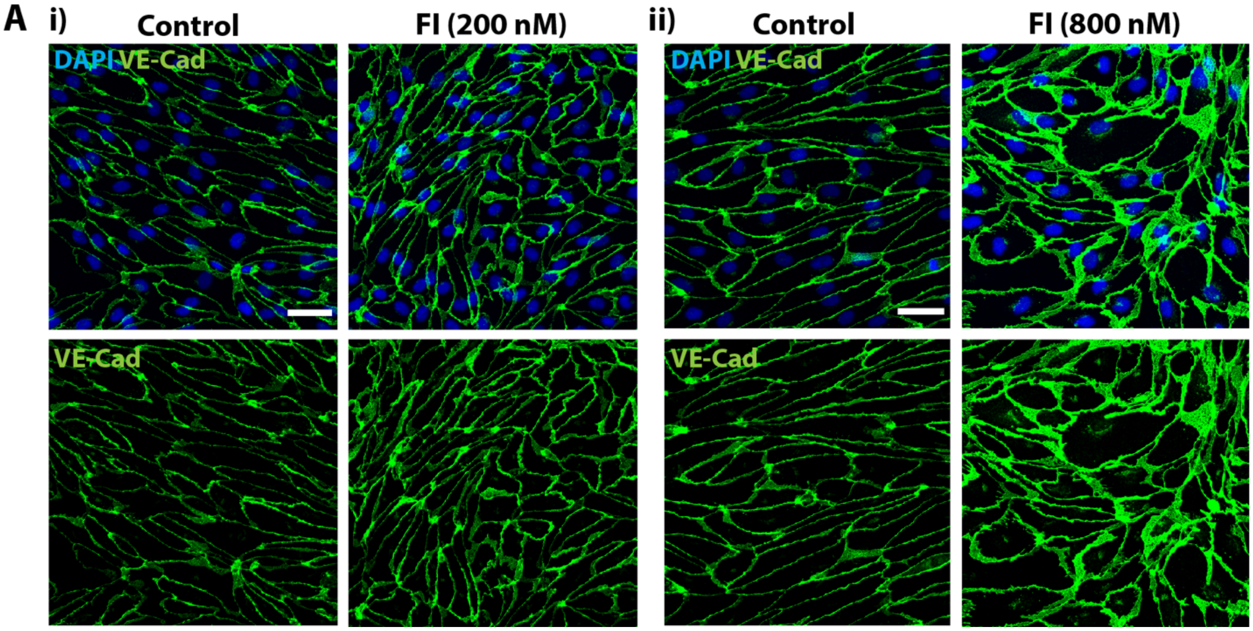

Supplementary Figure 6. FAK inhibition increases adherens junction stability. A. Representative images of EC monolayer incubated with 200 nM and 800 nM of FAK inhibitor show an increase in adherens junction size upon treatment (DAPI in blue and VE-Cad in green). Scale bar is 100 µm.
